# Pinpointing gravitational waves via astrometric gravitational wave antennas

**DOI:** 10.1038/s41598-024-55671-9

**Published:** 2024-03-01

**Authors:** Mariateresa Crosta, Mario Gilberto Lattanzi, Christophe Le Poncin-Lafitte, Mario Gai, Qi Zhaoxiang, Alberto Vecchiato

**Affiliations:** 1 Astrophysical Observatory of Turin (OATo)-INAF, via Osservatorio 20, 10025 Pino, Torinese Italy; 2 SYRTE, Observatoire de Paris, Université PSL, CNRS, Sorbonne Université, LNE, 61 avenue de l’Observatoire, 75014 Paris, France; 3 Chinese Academy of Sciences (CAS), Shanghai Astronomical Observatory (SHAO), 80 Nandan Road, Shanghai, 200030 China

**Keywords:** Astronomy and astrophysics, Astronomical instrumentation, General relativity and gravity, Stars

## Abstract

The direct detection of gravitational waves by ground-based optical interferometers has opened a new window in astronomy. Besides, the sensitivity of these linear detectors to the direction of arrival of an incoming gravitational wave is limited compared to current prospects of high-precision, space-based, astrometry. Indeed, advanced methods of differential relativistic astrometry offer a unique opportunity to overcome that situation. Here, we present a novel concept for a gravitational wave antenna that uses angles between close pairs of point-like sources as natural (angular) “arms” to characterise the very tiny variations in angular separations induced by a passing gravitational wave. The proposed new astrometric gravitational wave observable proves to be a powerful tool to substantially enhance the effect of gravitational waves of different strengths by exploiting optical resolution to the fullest. Then, by optically multiplexing three (or more) of such astrometric “arms”, it would be also possible to pinpoint source directions to unprecedented levels.

## Introduction

Experimental confirmation of gravitational waves (GWs) through the LIGO^1,2^ and VIRGO^3^ antennas has gained great impulse to the search and characterization of candidate GW sources. New ground-based experiments worldwide are already^4^, or in the process of^5–7^, joining the effort, and the LISA mission^8^ will soon implement similar concepts in space. The prime objective of this effort is the complete characterization of GWs, i.e., to determine their amplitude and frequency spectra, and then pinpoint their directions for multi-wavelenght “profiling”. A review on gravitational-wave physics and astronomy in the present decade can be found in Ref.^9^.

Astrometric (angular) observations are generally targeted for the accurate determination of directions to the incoming photons and their change with time. Therefore they collect photons that have interacted with different time-dependent gravitational fields along their path to the observer. With the advent of highly accurate astrometric and radial velocity measurements in space, observation reduction models compliant with General Relativity (GR) have become a necessity. As, when compared to the targeted measurement precision levels, the relatively small amount of space-time curvature due to the Sun and all of the other relevant Solar System (SS) masses (including the Earth-Moon system) affecting incoming photons and space-borne “observers” alike can no longer be ignored. The weak gravity regime influences electromagnetic propagation on a much wider domain than its strong counterpart.

Gravitational wave detection via astrometry was explored by some authors^10–15^. These authors consider the extra-shifts on a single light direction induced by passing GWs, i.e., on photons propagating to an observer from within the SS. Such a detection is not considered promising, since it requires at least nanoarcsecond (nas) accuracy^16–18^, and demands knowledge of satellite attitude to similar levels. Also, initial investigations on the potential of the Gaia astrometry in GW research focused on periodic GW signals with period shorter than the Gaia operating time^19,20^, or on secular effects^21^ on QSO proper motions for longer periods, i.e. for ultra-low GW frequencies of cosmological nature.

As a matter of fact, the astrometric observable considered so far, like in the case of Gaia, is expressed as the direction cosine between incoming stellar light and the observer attitude-tetrad^22^. Its strict application to a passing GW, being the tiny angular variations to stellar directions in the argument of the direction cosines, imposes a very strong requirement on the knowledge of satellite attitude at, or below, the microarcsecond ($\mu$as) accuracy, beyond current feasibility.

Disentangling GW signals also requires that the SS background metric is consistently developed to account for possible background (natural) systematics, thus preventing unwanted effects in modelling our astrometric observable. With $v/c \sim 10^{-4}$ rad, *v* being the typical velocity of the relevant SS metric sources, terms in the null geodesic solution should be retained to $v/c \sim 10^{-4}$ at least, i.e., below the nano-arcsec (*nas*) level^18^.

To overcome the above limitations, here we present a novel idea of an ${Astrometric~ GW~ Antenna}$ that fully exploits a GR formulation for differential astrometry, i.e., uses as antenna arms angles between close stellar pairs as measured at the observer’s location.

We first introduce a different fundamental observation equation for the astrometric GW detection based on sufficiently narrow angles between double stellar-like sources each materializing pairs of local lines of sight (LOS’s). We then give a first evaluation of its potential impact on GW science and its practical feasibility, in the light of new, specialized and much improved concepts for space astrometry missions^23–25^.

## Results

### The fundamental observation equation for the astrometric gravitational wave antenna

Let us assume the global metric due to both SS sources and passing or standing GW perturbations at the observer’s location in the form

1 $$\begin{aligned} { g}_{\alpha \beta }= {g}^{SS}_{\alpha \beta } + {h}_{\alpha \beta }^{GW} = \eta _{\alpha \beta } + \sum _{(a)} {h}_{(a) \alpha \beta }^{SS}+ {h}_{\alpha \beta }^{GW} + O(h^2) ~, \end{aligned}$$

where $\eta _{\alpha \beta }$ is the flat Minkowskian metric with signature $(-,+, +,+)$ and the subscript (a) stands for the a-source. The cosine of the angle between two observed (*obs*) light directions ${\ell }^{\alpha }_1$ and ${\ell }^{\alpha }_2$ writes (see Methods for the derivation)

2 $$\begin{aligned} \cos \psi _{1,2} = {g}_{\alpha \beta } ({ \ell }^{\alpha }_1 { \ell }^{\beta }_2 )_{obs}~, \end{aligned}$$

where ${ \ell }^{\alpha }$ is the null tangent unit four-vector projected on the rest space of the local barycentric observer, namely, for of our SS, the observer at rest relative to the barycentric celestial reference system (BCRS) with coordinates $(t,x^i)$. Definition (2) guarantees that $\psi _{1,2}$ is an observed quantity; its differential nature greatly relaxes precision requirements on the knowledge of satellite attitude (see comments in Methods) and payload (thermal and mechanical) stability.

Similarly to what just done for the metric, stellar light directions can be represented as the SS part (due to the background metric) plus a perturbation shift, i.e. $\delta \ell ^{\alpha }$, attributed purely to the passing GW:

3 $$\begin{aligned} {{\ell }}^{\alpha }_{obs} = \ell ^{\alpha (SS)} + \delta \ell ^{\alpha } + O( \delta \ell ^2), \end{aligned}$$

in particular for the SS part

4 $$\begin{aligned} {\ell }^{\alpha (SS)}_{obs} = {\ell }^{\alpha }_{_0} + \epsilon { \ell }^{\alpha }_{_{(1)}} + \epsilon ^2{\ell }^{\alpha }_{_{(2)}}+ \epsilon ^3 {\ell }^{\alpha }_{_{(3)}}+ \epsilon ^4 { \ell }^{\alpha }_{_{(4)}} +O( \epsilon ^5), \end{aligned}$$

where $\epsilon = v/c$, being *v* the typical velocity of each relevant SS metric source, and the subscripts in parenthesis indicate the order of approximation. Then, the right-hand side of (2) can be further simplified, assuming $\delta \ell$ starts at $\sim \epsilon ^4$, as

5 $$\begin{aligned} \cos \psi _{1,2} =\cos \psi _{1,2}^{SS} + \eta _{\alpha \beta } ({\ell }^{\alpha }_{1_0} \delta \ell _{2}^{\beta } + {\ell }^{\alpha }_{2_{0}} \delta \ell _1^{\beta })+ h^{GW}_{\alpha \beta } {\ell }^{\alpha }_{1_0} {\ell }^{\beta }_{2_{0}} + O(\epsilon ^5)+ O(h^2), \end{aligned}$$

${\ell }^{\alpha }_{i_0}$ representing the unperturbed (Minkowskian) light direction to star i=1,2. Alternatively, (5) can be reformulated as

6 $$\begin{aligned} \cos \psi _{1,2} = \cos \psi _{1,2}^{SS} + F^{GW}_{1,2}, \end{aligned}$$

where we collect all the GW terms related to the theoretical modelling in the expression $F^{GW}_{1,2}\equiv \eta _{\alpha \beta } ({\ell }^{\alpha }_{1_0} \delta \ell _{2}^{\beta } + {\ell }^{\alpha }_{2_0} \delta \ell _1^{\beta })+ h^{GW}_{\alpha \beta } {\ell }^{\alpha }_{1_0} { \ell }^{\beta }_{2_0} + O(\epsilon ^5)+ O(h^2)$. Note that the right-hand side of Eqs. (5) and (6) is a spacetime invariant that can be evaluated in any coordinate system.

On the other hand, since the passing GW produces an extra shift on light deflection, we expect a perturbation $\delta \psi ^{GW}$ to the undisturbed angle between two light directions.

Then, we can expand the left-hand side of our observation Eq. (2) (i.e., its observational part) as follows:

7 $$\begin{aligned} \cos (\psi _{1,2}^{SS} + \delta \psi _{1,2}^{GW}) = \cos (\psi _{1,2}^{SS}) \cos ( \delta \psi _{1,2}^{GW}) - \sin (\psi _{1,2}^{SS}) \sin ( \delta \psi _{1,2}^{GW}). \end{aligned}$$

Considering, now, both Eqs. (6) and (7), we obtain

8 $$\begin{aligned} \cos (\psi _{1,2}^{SS}) \cos ( \delta \psi _{1,2}^{GW}) - \sin (\psi _{1,2}^{SS}) \sin ( \delta \psi _{1,2}^{GW}) = \cos \psi _{1,2}^{SS} + F^{GW}_{1,2}. \end{aligned}$$

Since $\delta \psi ^{GW}\ll 1$ and for $\delta \psi ^{GW} \ll \psi ^{SS}$, the previous equation reduces to consider the unknown angle due to the passing GW, i.e.

9 $$\begin{aligned} \delta \psi _{1,2}^{GW} = - \frac{F^{GW}_{1,2}}{\sin (\psi _{1,2}^{SS})}. \end{aligned}$$

The new expression (9), deduced from observation Eq. (2), provides the observed perturbation to the $\psi _{1,2}^{SS}$ angle caused by the passing GW in the $F^{GW}_{1,2}$ term. Angular perturbations can be made larger through the factor (sin$(\psi _{1,2}^{SS}))^{-1}$ that acts as a “signal amplifier” for the GW detection. That this amplification factor is not arbitrary is clearly a consequence of the condition $\delta \psi ^{GW} \ll \psi ^{SS}$ and the fact that (see below for further clarification on this point) an optical device, a telescope, implementing Eq. (9) has to be able to measure the angular distance of the 1,2 star-like pair materializing one arm of the astrometric antenna, and this is mainly limited by its resolving power.

Terms like the $1 / \sin (\psi )$ factor are rather typical when the effects of gravitational perturbations need to be put to test as this is accomplished by utilizing measurements of angles between directions to pairs of stellar-like objects. An example is that in, e.g.,^26^ where, after modeling the deflection of light from a target source due to a spherical mass along the line-of-sight, the Authors turn the discussion into the actual way of measuring the effect by using the angle $\theta$ between the target and a reference source, a direct analogue of our angle $\psi$ between two sources forming one arm of the antenna. As, in close analogy with linear GW antennas, the angle $\psi$ represents one ${angular}$ arm of our proposed astrometric GW antenna. It is by increasing the length L of the arms of linear antennas that the effect of the GW perturbation becomes easier to measure through the relation $\delta L \sim h^{GW}~ L$; analogously, it is by being able to measure smaller and smaller separation angles (i.e., increasing the resolution power of the optics used) that we can increase the measurability on the GW-induced effect on an (angular) arm of the astrometric antenna via our relation (9), i.e., $\delta \psi \sim h^{GW} / \sin (\psi )$.

As we are putting forth a novel operational principle for measuring GWs that takes great advantage from the quantity $\psi ^{SS}_{1,2}$, here it will suffice to indicate how that can be estimated/derived from actual measurements of the pair separation. What we measure is actually the angle $\psi _{1,2}(t_i)$ between point-like sources ’1’ and ’2’ at time $t_i$. The measurements $\psi _{1,2}(t_i)$ are taken with high cadence, i.e., with frequency $\omega _{S} \gg \omega _{GW}$, the oscillating frequency of the GW (assumed ’monochromatic’ ) we seek to unveil. This last condition ensures that the Nyquist–Shannon (sampling) theorem is satisfied and, at the same time, sufficient statistics is built to beat (single) measurement noise.

Considering the average $< \psi _{1,2}(t_i)>_N$ over the N separations $\psi _{1,2}(t_i)$ taken over a measurement session, with N $\gg$ 1, we have: $< \psi _{1,2}(t_i)>_N$ $=< \psi ^{SS}_{1,2}>_N + < \delta \psi ^{GW}_{1,2} (t_i) >_N$ $\simeq \hat{\psi }^{SS}_{1,2}$, as we can make the average $< \delta \psi ^{GW}_{1,2} (t_i) >_N$ as small as needed, much like it is done in signal spectral analysis. And, with this evaluation of the unperturbed angular separation of our antenna arm (the point-like pair) directly from the observations it follows that: $\delta \hat{\psi }^{GW}_{1,2} (t_i) \equiv \psi _{1,2}(t_i) - \hat{\psi }^{SS}_{1,2}$.

This is, in principle, how the GW perturbation to the antenna angle can be estimated directly from the measurements, along with $\hat{\psi }^{SS}_{1,2}$, and then used in Eq. (9) to build the observation equations from which strength and direction to the GW source can be recovered.

### The operating principle of the GW astrometric antenna

The measurement and initial data processing protocol just sketched supports, at least in principle, the practicability of our concept for an astrometric GW antenna.

To provide quantitative examples suggestive of the potential of Eq. (9), it will suffice to consider a plane GW, of a given frequency $\omega_{GW}$, linearly polarized (i.e., $A_{\times }=0$, see below). Indeed, the actual application of Eq. (9) requires the definition of the following ingredients: (1) the $h^{GW}$ at the observer, (2) a pair of unperturbed local lines-of-sight, $\ell ^{\alpha }_{i_0}$ (see Eq. 3), and (3), the corresponding shift $\delta \ell$ due to the passing GW from the geodesic equation with metric (1).

The general form of the GW perturbation $\delta \ell$ can be expressed as a function of argument ${\tilde{k}}_{\alpha } x^{\alpha }$, namely $h_{ij}^{GW}({\tilde{k}}_{\alpha } x^{\alpha })$, with tangent vector ${\tilde{k}}^{ \alpha } = {\tilde{k}}^0 \partial _0^{ \alpha } + {\tilde{k}}^i \partial _i^{ \alpha },$ where $p^i = {\tilde{k}}^i/ {\tilde{k}}^0$ is the direction of the GW propagation. Then, in the linearized regime, the gravitational wave shift is recovered within the suitable astrometric models, via the geodesic integration with respect to a suitable parameter $\sigma$ (see “Methods” for details), namely:

10 $$\begin{aligned} \int ^{\sigma _*}_{\sigma _{obs}} d \delta \ell ^i = \frac{ \ell ^i_{_0} + p^i}{2 (1 + p \cdot \ell _{_0})} \ell ^j_{_0} \ell ^k_{_0} \int ^{\sigma _*}_{\sigma _{obs}} d h_{jk}^{GW} (\sigma ) - \frac{\ell ^j_{_0}}{2} \int ^{\sigma _*}_{\sigma _{obs}} d h_{ij}^{GW} (\sigma ) + O(\epsilon ^5), \end{aligned}$$

that, in the far away zone, reduces to

11 $$\begin{aligned} \delta \ell ^i = \frac{ \ell ^i_{_{0}} + p^i}{2 (1 + p \cdot \ell _{_0})} \ell ^j_{_{0}} \ell ^k_{_{0}} h_{jk}^{GW}(\sigma _{obs}) - \frac{ \ell ^j_{_{0}}}{2} h_{ij}^{GW}(\sigma _{obs}) + O(\epsilon ^5), \end{aligned}$$

explicitly showing that $\delta \ell ^i \propto h^{GW}$, and in agreement with what found in Ref.^11,14^. In such a case, our expression (9) becomes

12 $$\begin{aligned} \delta \psi _{1,2}^{GW}= & {} - \frac{ h^{GW}_{ij}(\sigma _{obs})}{2 \sin (\psi _{1,2}^{SS}) } \left\{ \frac{[( \ell _{1_0} \cdot \ell _{2_0}) + ( \ell _{1_0} \cdot p) ] \ell ^i_{ 2_0} \ell ^j_{ 2_0}}{(1 + p \cdot \ell _{2_0})} + \frac{ [( \ell _{2_0} \cdot \ell _{1_0}) + ( \ell _{2_0} \cdot p) ] \ell ^i_{ 1_0} \ell ^j_{ 1_0}}{(1 + p \cdot \ell _{1_0})} \right\} \nonumber \\{} & {} + O(\epsilon ^5), \end{aligned}$$

which shows also the dependence on the scalar products between the SS Minkowskian directions of photon propagation from stars 1 and 2 (i.e., alternatively, the angle $\psi _{1_0, 2_0}$) and on the scalar products of each star direction to the GW source (angles $\psi _{1_0, p}$ or $\psi _{2_0, p}$).

Lastly, it is worth stressing once more that Eq. (9) holds only for $\delta \psi ^{GW}<< \psi ^{SS}$. Increasing the optical resolution power to infinity, i.e., for the pair separation angle $\psi$ going to zero, would make our observable $\delta \psi ^{GW}$ degenerate. This can be easily seen from Eq. (6) where, with $\psi$ $\rightarrow 0$, the two cosine terms on both sides of this equation $\rightarrow 1$, leaving $F^{GW}=0$. As $F^{GW}$ does not depend on $\psi$ (Eq. 12), this can only mean that our observation Eq. (6) through Eq. (9) (or Eq. 12) degenerate and would no longer be useful to measure the effect of a passing $h^{GW}$.

Equation (12) above governs the operating principle of (one angular arm) of the astrometric antenna, and clearly shows its direct relation with the direction to an incoming GW. For, the GW term $h_{ij}$ (and its time variation) will mostly characterize the detection (amplitude and phase term), while the factor within the curly brackets will assume specific patterns according to the direction of the incoming GW relative to the observer (spatial) orientation.

### Pinpointing sources of gravitational waves

**Figure 1:**
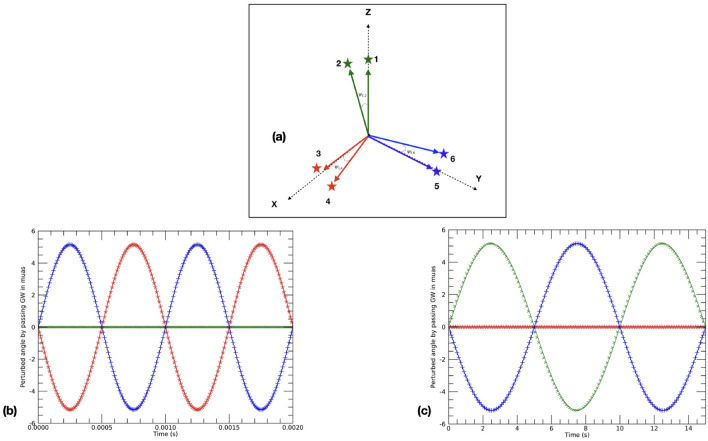
In (**a**) a possible configuration of a 3-LOS telescope, the ($\textbf{x}$,$\textbf{y}$, $\textbf{z}$) triad, with respect to the chosen directions: the local (SS) vectors $\ell _{3_0}$, $\ell _{5_0}$ and $\ell _{1_0}$ to stars 3,5 and 1, respectively. The angles ${\psi }_{i, j}$, representing the instantaneous angular distances of the stellar pairs are all assumed small at $\sim 0.01''$ for (**b**) and $\sim 0.001''$ for (**c**). With the same colour coding, the other two panels show, fixed the resolution limit, the astrometric signals $\delta \psi ^{GW}_{i,j}$ (from Eq. (12)) caused by a GW passing along: (**b**) the $\textbf{z}$ direction (green line) with $A_{+}\sim 10^{-18}$ and frequency $10^3 Hz$; (**c**) the $\textbf{x}$ direction (red line) with amplitude $A_{+}\sim 10^{-19}$ and frequency 0.1 *Hz*. The other curves show the perfect anti-correlation of the GW-induced signals along the other two LOS’s of the astrometric antenna.

**Figure 2:**
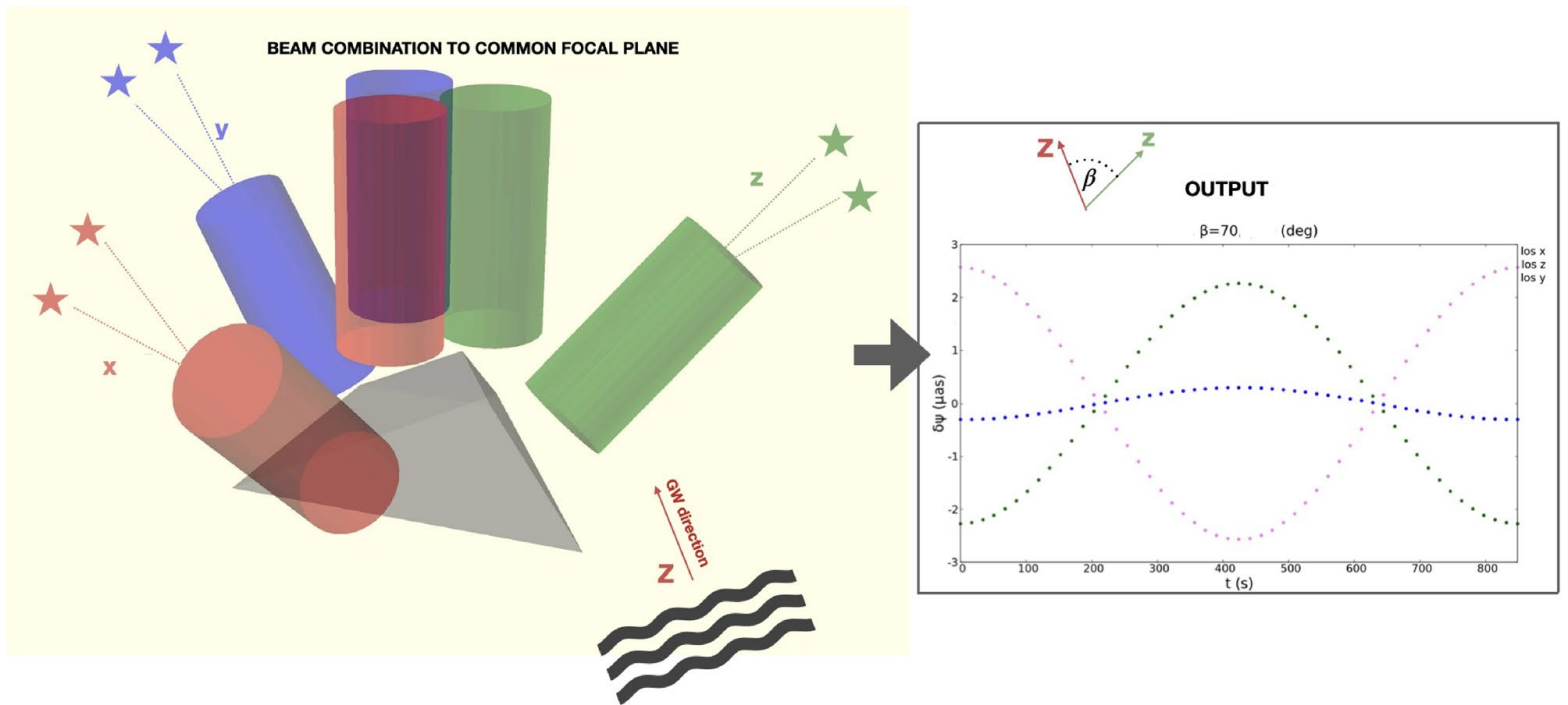
Schematic illustration of a 3-LOS telescope endowed with a beam combination module on a common focal plane. Differently from Fig. 1a the GW direction Z is 70$^{\circ }$ from the z axis of the ($\textbf{x}$,$\textbf{y}$, $\textbf{z}$) triad. The output contemplates the case reported in the forth row of Table 1 with $\omega \sim 10^{-3}Hz$ and $A\sim 10^{-19}Hz$ for ${\psi }_{i, j} \sim 0.01''$ . The minimum signal (the blue dots) points towards the GW direction.

The spatial orientation of the observer, i.e., an optical system endowed with three viewing directions (or lines of sight-LOS) multiplexed onto a common focal plane, is idealized in Fig. 1a as a ${{\textbf{x}}}, {{\textbf{y}}}, {{\textbf{z}}}$ triad and schematically illustrated in Fig. 2.

**Table 1:** Angular perturbations, $max(\delta \psi ^{GW}_{i,j})$ ($\mu$as), for different linear strains of amplitude $A_+$ (radians) propagating along ${{\textbf{z}}}$. The values derive from Eq. (12) after setting the three antenna arms $\psi _{i_0,j_0}$ to the values in the second column (in arcsec). As expected, decreasing $\psi _{i_0,j_0}$ by one order of magnitude, the corresponding $\delta \psi ^{GW}_{i,j}$ is amplified by the same amount. This is the case for the values reported in the second row compared to its direct analogue in first. Note that along the GW direction of propagation $\delta \psi ^{GW}_{1,2}$ is not null, as the direction to star 2, forming the 1-2 pair, although quite small, is not coincident with the LOS to star 1.

$A_{+}$ (radians)	$\psi _{i_0,j_0}$ (arcsec)	$max(\delta \psi ^{GW}_{1,2})$ ($\mu$as)	$max(\delta \psi ^{GW}_{3,4})$ ($\mu$as)	$max(\delta \psi ^{GW}_{5,6})$ ($\mu$as)
$10^{-18}$	0.01	$4.85 \times 10^{-15}$	4.85	4.85
$10^{-18}$	0.001	$4.85 \times 10^{-16}$	48.57	48.57
$10^{-19}$	0.01	$4.85 \times 10^{-16}$	0.49	0.49
$6\times 10^{-19}$	0.01	$3.02 \times 10^{-15}$	2.57	2.57
$10^{-20}$	0.01	4.85$\times 10^{-17}$	4.85 $\times 10^{-2}$	$4.85 \times 10^{-2}$
$10^{-21}$	0.01	0	$4.85 \times 10^{-3}$	$4.85 \times 10^{-3}$

The local (SS) unperturbed direction to star 1 is along the same direction at which LOS ${{\textbf{z}}}$ is pointing. Also, $\ell _{ 2_0}$, the local direction to star 2, is at a very small angular separation to $\ell _{ 1_0}$, i.e., $\cos (\psi _{1_0,2_0}) \sim 1$.

Similarly to the LOS in the ${{\textbf{z}}}$ direction of our schematic 3-way telescope, one can imagine to have the other two viewing directions, ${{\textbf{x}}}$ and ${{\textbf{y}}}$, aligned along the local directions $\ell _{3_0}$ and $\ell _{5_0}$ to stars 3 and 5, respectively (Fig. 1a), while directions $\ell _{4_0}$ and $\ell _{6_0}$, to stars 4 and 6, are the corresponding close optical companions. Close stellar (or stellar-like) pairs here mean that we are always concerned with angles ${\psi }_{i_0, j_0}$ $\lesssim$ 0.01 arcsecond, a number representative of the operational resolution limit reached with telescopes already operating (e.g. HST, ESA’s Euclid) or that will soon operate in space (like, e.g., NASA’s NextGen Space Telescope, CNSA’s CSST or see further below for more) at optical wavelengths, i.e. $\ge 550$ *nm*, including the near-IR (to $\sim 2$ micron).

In the “Transverse and Traceless” (TT) standard gauge the GW components are $h_{0 i}=0$, $\delta ^{ij} h_{ij}=0$, $\delta ^{ij} h_{jk,i}=0$, with only two independent degrees of freedom, the two amplitudes $A_{+}$ and $A_{\times }$. Taking advantage of the property that in the TT gauge only the components perpendicular to the direction of propagation survive, we proceed to show, without loosing too much to generality, the principle of astrometrically measuring a GW reaching a space-born observer from within the SS, including its direction.

We utilize representative cases of GW strains known from the literature. The first column of Table 1 presents the amplitude, $A_{+}$, of metric perturbations from possible GW sources as described in Refs.^27,28^. The last three columns provide, for each $A_{+}$, the maximum (angular) perturbations resulting on the local unperturbed (angular) separations of the pairs (the “arms” of the astrometric antenna) along the three telescope axes (Fig. 1a). The perturbation signals can be calculated from Eq. (12) for a plane GW traveling along the ${{\textbf{z}}}$ direction and ${\psi }_{i_0, j_0}$ set to the values in Table 1. Gravitational strains as large as $h \sim 10^{-18}$ are associated with SN core collapse events, thus, they are short lived and at frequencies $\omega \sim$ 10$^3$ Hz. To the range of high frequency sources, $\omega$=[10 – 1,000] Hz, belong also the cases of coalescing compact binary systems (NS-NS, pairs of stellar black holes, BH$^*$-BH$^*$, or NS-BH$^*$). Besides, at $\omega<$ 1 Hz and in a range of characteristic strain amplitudes spanning 3 orders of magnitude, from $\sim$ 10$^{-18}$ to 10$^{-21}$, one finds not only coalescing super-massive BH’s, but also the significantly more numerous population of resolved and unresolved Milky Way binaries, with at least one degenerate companion. The fourth row in Table 1 reports, as an example, the output of a linearized GW signal produced by an hypothetical binary system, at the distance of 100pc, with masses $m_1= 20 M_{\odot }$ and $m_2 =15 M_{\odot }$, an orbital separation of 1 $R_{\odot }$, and frequency $\omega \sim 10^{-3} Hz$.

Figure 1b shows 5 ms (i.e. 5 times the simulated period) of the angular perturbation $\delta \psi ^{GW}_{i,j}$ experienced by the three ”arms” (the angles ${\psi }_{i_0, j_0}$) under the strain of the high frequency-high amplitude case mentioned before. As expected, with a GW propagating in the direction of the ${{\textbf{z}}}$-axis, the 1–2 stellar pair is practically unperturbed, while all the action is with the pairs along the $\textbf{x}$ and $\textbf{y}$ axes.

If statistically meaningful measurements of $\sim 5 \mu as$ variations in angular separations, as in Fig. 1b, although possible, are yet to be proven, variations 10 times larger are already within the capabilities of present-day, or soon-to-be, space astrometry missions, like those mentioned earlier (see Ref.^23^ and references therein). This is possible also because actual (angular) resolution limits, governing the amplifications of the perturbation signal associated with a GW (Eq. 12), can be made significantly higher by utilizing optimal calibrations procedures^29–31^. Signal frequencies of 1 kHz, as in Fig. 1b are hard to achieve with high signal-to-noise ratio, as sampling above the Nyquist-Shannon frequency would call for integration times shorter than 0.5 *ms*. ”Fast” exposure times are at 25 ms in the case of the astrometer FGS aboard HST^32^ (and references therein), and only 100 ms on Euclid^33^ (and references therein), clearly not enough. And, simply allowing for much shorter exposure times might not improve things as, given the size of the telescope mirrors on these missions, it might become difficult to reach sufficiently faint magnitudes, thus forcing the 3-LOS telescope to limit its orientations to directions with sufficiently bright stellar pairs.

Besides, as mentioned earlier, GW sources with similar amplitudes, or even larger, are expected at more comfortable frequencies, making this scenario a clear case for GW science with an astrometric antenna.

Figure 1c illustrates the progress of $\delta \psi ^{GW}_{i,j}$ with time for the GW strain amplitude in row 2 of Table 1; $\omega =$ 0.1 Hz, while the separations of the angular arms are here set to 0.001$''$, instead of 0.01$''$, thus generating variations of a few $\mu$as. Therefore, this figure and the considerations on the cases presented in Table 1 confirm that an astrometric antenna, capable of monitoring periodic signals of amplitude > 1 $\mu$as, would ideally possess the ability of measuring GW’s associated with a range of coalescing massive BH’s events.

Finally, the last two rows of Table 1 refers to smaller amplitudes as expected for GW’s from core collapse events (at $\omega \sim 100$ Hz), or, at much shorter frequencies, from resolved and unresolved MW binaries. These cases appear beyond today’s technology on current missions, including payloads that will be flying into orbit within the next few years. Therefore, further expanding the access to the physics of GW’s for an astrometric antenna would require dedicated technological developments (see e.g., Refs.^23,34^).

The priority of this article was to prove the concept of a novel idea that might develop into new space-borne astrometric instrumentation for the study of GW’s. From this perspective, it is evident that the angular arms of a 3-LOS GW antenna, as schematically depicted in Fig. 1a, would register perfectly correlated astrometric signals with amplitudes and phases depending on actual orientations of incoming GW’s. For the ideal examples in Fig. 1b,c, these data would immediately tell the direction of arrival of the gravitation strain causing the coherent “trembling” in the antenna arms (the chosen stellar pairs) as that of minimum signal. Therefore, the ultimate precision with which a direction can be recovered is sub-*mas*, independently from when an actual implementation of our astrometric antenna concept might be sent into orbit, as the sources will be in the Gaia Catalogs (see, e.g., Ref.^35^), and therefore their absolute coordinates known to high accuracy. Or, “worst case”, one could say the actual directional uncertainty is comparable to the angles separating the stellar pairs used as arms, i.e., $\sim$ 10 mas or less. Either case, these are unprecedented numbers and pinpointing source directions to such accuracies would tremendously help multi-wavelength and multi-messenger follow-up investigations.

As for the stability of the LOS’s (often referred to as telescope pointing stability) during elementary photon integration times (individual exposures), this enters the error budget at the level of the elementary angular separations measurements of the pairs, each materializing an astrometric antenna arm. Assuming the pointing stability as a random process, its effect adds in quadrature to the angular size of an (aberration free) optical diffraction pattern of the point-like sources forming each pair. Pointing stabilities of 1 mas can be routinely achieved on modern space observatories (see, e.g., ^30^, and references therein, for the HST), and this must be compared to diffractions patterns of ∼ 60 mas for 2m circular apertures at λ∼ 600 nm, therefore contributing a negligible effect. Besides, even in less ideal conditions, the differential nature of our fundamental measurements is such that pointing errors are minimized as common mode effect on angular separations, especially at the resolutions of interest here.

## Discussion

Immediately after the initial development at the idea of an antenna for measuring GW’s using astrometry from a telescope in space (see sec. 6.4 in Ref.^36^), we began efforts to prove, with simulations and laboratory tests, the feasibility of an astrometric antenna based on the precepts described in this article (Refs.^24,25,37^ and references therein). We refer to those studies for results and discussions on what can be currently said on implementation issues like: (1) the very possibility to build a 3-LOS multiplexing telescope (Ref.^38^ and references therein), (2) the limit of centering accuracies of star-like images on digital detectors, (3) actual (beyond Rayleigh’s) resolution limits for the antenna arms (depending not only on optics and detection system, but also on magnitude and color of the stellar pairs), (4) other natural (intrinsic or cosmic) causes of astrometric noise as stellar activity, and (5) identify (via spatial laser metrology of critical degrees-of-freedom) and deal with instrumental noise mimicking unwanted variations of the antenna arms $\psi _{i_0,j_0}$.

To date, before any attempt at extracting GW signals from Gaia-like astrometric data, one would generally consider the end of the global reduction process in order to obtain the best possible knowledge of satellite attitude (orientation) and instrumental behaviour at all of relevant time scales. In such a context, looking for variations in the direction to a single source on the sky at the *nas* ($\sim 5 \times 10^{-15}$ rad) level implies the knowledge of an “absolute” reference, e.g. the telescope LOS, at comparable precision $\sim 10^{-15}$. This is an impossibile requirement on the reconstructed attitude of a science satellite. The differential technique proposed in Refs.^24,25^ aims at *nas* measurement over a distance of the order of 1”, thus implicitly reducing the relative precision requirement to $\sim 10^{-9}$, with an improvement of six orders of magnitude. Also, instrument calibration requirements are strongly alleviated. In Gaia, we have a variation of the electro-optical response of hundreds of mas over the $0.5^\circ$ field, calibrated to the $\mu$as level.

Assuming a linear model, the corresponding electro-optical response variation for an astrometric GW telescope over 1” would be in the range of hundreds of $\mu$as, i.e., a comparable calibration “power” would scale the measurement reliability to the *nas* regime. Actually, optimal optics design, exploiting the higher-than-linear decrease of many aberrations close to the optical axis, would reduce instrumental contribution even further^37^.

In addition to the above, we will have to simulate much more realistic scenarios (i.e., more general forms of GW’s and use real-sky pairs) and conditions (realistic noise levels) to investigate viable strategies for the actual retrieval of amplitude and phase (carrying the direction information, Figs. 1b,c and 2). However, especially for these aspects, help would certainly come from the large amount of work done, and proven on real data by the LIGO and VIRGO collaborations.

Nevertheless, as we show in this work, the astrometric observable could amplify the GW-induced signal if one takes into account the angle between two space-like directions of light^36^ in the framework of general relativistic astrometry (see e.g. Ref.^22^). In such a case, one is in principle exempted from dealing with satellite’s attitude and the GW astrometric measurement can be translated into an observation equation accounting for a wide range of frequencies.

The diversity of GW frequencies that can potentially be treated with Eq. (12) and appropriate modeling of gravitational shifts could improve PTA and/or LISA observations^8,39^, and the low frequency domain due to periodic sources (e.g., Galactic binary WDs identified by Gaia), thus further enhancing the mapping of the Milky Way substructures^40^. Moreover, the advantage of Eq. (9) is the possibility to exploit a large number of null geodesics, so to better scrutinize the GW direction, a critical aspect of the GW detection and multiwavelength characterization. This same feature would also enable tests on GW polarization modes by combining different telescope orientations.

Finally, the same principle of a space-born multi-LOS telescope discussed here can push investigations on possible ground-based realisations of an astrometric GW antenna. Such a development could support present and future linear interferometer by working as signal sentinel and by spotting almost immediately directions to incoming signals, thanks to the fully correlated nature of the GW-induced astrometric signals expected from the combined LOS’s (Figs. 1b,c, and 2).

In conclusion, the potential of the relativistic astrometric observable advocated here for the astrometric detection and precise identification of gravitational waves, by using pairs of natural stars, will significantly add to the best GW detection procedures in use. In fact, the availabiltiy of close stellar pairs opens to the exploitation of a large number of configurations, and therefore to monitoring gravitational waves coming from any direction; thus providing, at the same time, extensive statistics to uncover the properties of a GW source. This helps in a truly complementary and independent way all of the efforts dedicated to multiband GW searches bridging low and high frequencies at different redshifts (see, for example, Refs.^41–43^). And, in case the two unperturbed LOS’s of a single astrometric antenna arm, although angularly very close, are actually related to two stars at different distances, it would be also possible to investigate time retarded effects and test GW speed. Last, a suitable choice of the strain $h^{GW}$ could pave the way for new GW tests on gravitation interaction with photons: it would suffice modeling the $F^{GW}$ function appropriately.

## Methods

### The cosine expression for the GW observation equation

The cosine of two light directions (i,j) is defined in General Relativity as:

13 $$\begin{aligned} \cos \psi _{i,j} = \frac{P(u)_{\alpha \beta } k^\alpha _i k^\beta _j}{(\sqrt{ P(u)_{\alpha \beta } k^\alpha _i k^\beta _i}) (\sqrt{ P(u)_{\alpha \beta } k^\alpha _j k^\beta _j} ) } \end{aligned}$$

where $P(u)_{\alpha \beta }= g_{\alpha \beta } + u_\alpha u_\beta$ is the operator that projects with respect to the local barycentric observer $u^\alpha = 1/\sqrt{-g_{00}}$. Then the photon 4-momentum can be decomposed as

14 $$\begin{aligned} k^{\alpha } =-(u|k)u^{\alpha } +l^{\alpha }, \end{aligned}$$

where $(u|k) = g_{\alpha \beta } u^{\alpha } k^\beta$ and $l^{\alpha }$ is the spatial null vector projected on the rest space of $u^\alpha$, Defining

15 $$\begin{aligned} {\bar{k}}^\alpha = -\frac{k^\alpha }{(u|k)} \,,\,\,\,\, \ell ^\alpha =- \frac{l^{\alpha }}{(u|k)}={\bar{k}}^\alpha -u^\alpha . \end{aligned}$$

the cosine for two directions i,j simplifies as

16 $$\begin{aligned} \cos \psi _{i,j} = {g}_{\alpha \beta } ({ \ell }^{\alpha }_i { \ell }^{\beta }_j )_{obs}. \end{aligned}$$

The BCRS metric is defined by IAU resolutions as a post-Newtonian (pN) solution of the Einstein field equations. Thus, one has to take into account terms of this metric accurate to the order of the GW perturbations sought for. Dropping the sum symbol, let us express the metric including both sources as:

17 $$\begin{aligned} {g}_{\alpha \beta }= \eta _{\alpha \beta } + \epsilon {h}^{SS}_{(1) \alpha \beta }+ \epsilon ^2 { h}^{SS}_{ (2) \alpha \beta }+ \epsilon ^3 { h}^{SS}_{ (3 )\alpha \beta }+ \epsilon ^4 { h}^{SS}_{(4) \alpha \beta }+ {h}_{\alpha \beta }^{GW} + O(\epsilon ^5) \end{aligned}$$

where $\epsilon$ is of the order of *v*/*c*, being *v* the typical velocity of each relevant SS metric source, and the subscripts in parenthesis indicate the order of approximation in $\epsilon$. We assume that the GW perturbations are of order $\epsilon ^4$ at best, i.e., at the nanoarcsecond level. Like for the SS metric, the SS contribution to light direction can be approximated as:

18 $$\begin{aligned} {\ell }^{\alpha (SS)}_{obs} = {\ell }^{\alpha }_{_0} + \epsilon { \ell }^{\alpha }_{_{(1)}} + \epsilon ^2{ \ell }^{\alpha }_{_{(2)}}+ \epsilon ^3 { \ell }^{\alpha }_{_{(3)}}+ \epsilon ^4 { \ell }^{\alpha }_{_{(4)}} +O( \epsilon ^5). \end{aligned}$$

From the assumptions above, one finally finds:

19 $$\begin{aligned} \cos \psi _{i,j} =\cos \psi _{i,j}^{SS} + \eta _{\alpha \beta } ({ \ell }^{\alpha }_{i_0} \delta \ell _{j}^{\beta } + { \ell }^{\alpha }_{j_{0}} \delta \ell _i^{\beta })_{obs}+ h^{GW}_{\alpha \beta } { \ell }^{\alpha }_{i_0} { \ell }^{\beta }_{j_{0}} + O(\epsilon ^5). \end{aligned}$$

### The total cosine versus an additive GW direction cosine

In this section we clarify the consequences, when in the presence of a passing GW, of utilizing the cosine as observable by simply extending what it is done in the context of the Gaia mission, where the direction cosine refers to the angle of the incoming light to the observer (satellite) attitude-tetrad $E^{\alpha }_{{\hat{a}}}$.

Let us consider the a priori assumption that the effect of a GW is that of adding a cosine term to the direction cosine associated with the SS metric, i.e., denoting with $\cos (\hat{\psi })$ the cosine of the angle $\hat{\psi }$ of a light direction to the tetrad, the total (tot) cosine is given by:

20 $$\begin{aligned} \cos (\hat{\psi })_{tot}= \cos (\hat{\psi })^{SS} + \cos (\hat{\psi })^{GW}. \end{aligned}$$

With the extra assumption that the correction $\delta \ell _{(GW)}$, induced by the GW to the SS line-of-sight at the observer $\ell _{(SS)}$, is (formally) known, Eq. (20) would read:

21 $$\begin{aligned} (\eta _{\mu \nu } + h^{SS}_{\mu \nu }) ( \ell ^{\mu }_{(SS)} E^{\nu }_{{\hat{a}}}) + (\eta _{\mu \nu } + h^{GW}_{\mu \nu }) (\delta \ell ^{\mu }_{(GW)} E^{\nu }_{{\hat{a}}}) \approx \cos (\hat{\psi })^{SS} + \eta _{\mu \nu } \delta \ell ^{\mu }_{(GW)} E^{\nu }_{{\hat{a}}}, \end{aligned}$$

implying no explicit dependence on the strain $h_{GW}$.

If, on the other hand, the GW perturbation is not a known part of the observed direction at the observer, one would have:

22 $$\begin{aligned}{} & {} (\eta _{\mu \nu } + h^{SS}_{\mu \nu }) [ (\ell ^{\mu }+\delta \ell ^{\mu }) E^{\nu }_{{\hat{a}}}] + (\eta _{\mu \nu } + h^{GW}_{\mu \nu }) [(\ell ^{\mu } + \delta \ell ^{\mu }) E^{\nu }_{{\hat{a}}}]\nonumber \\\approx & {} \cos (\hat{\psi })^{SS} + 2 \eta _{\mu \nu } \delta \ell ^{\mu } E^{\nu }_{{\hat{a}}} + \eta _{\mu \nu } \ell ^{\mu } E^{\nu }_{{\hat{a}}}+ h^{GW}_{\mu \nu } \ell ^{\mu }_{ 0} E^{\nu }_{{\hat{a}}}, \end{aligned}$$

with the flat Minkowskian metric term entering twice in the Equation for the $\ell ^{\alpha }$ component. Then, the simple addition to the “background” direction cosine in the SS metric (i.e., BCRS) would introduce the flat Minkowskian contribution twice without a priori disentanglement from the local-line-of-sight of the GW component or, if the GW shift is considered as a separate part, it would imply to discard a priori the $h^{GW}$ strain in the observation equation.

It is only when we drop the assumption made with Eq. (20), i.e. when working directly with the total cosine, that we finally recover an expression similar to observation equation in the Main article as:

23 $$\begin{aligned} \cos (\hat{\psi })_{tot}= (\eta _{\mu \nu } + h^{SS}_{\mu \nu } + h^{GW}_{\mu \nu })[ ( \ell ^{\mu } + \delta \ell ^{\mu }) E^{\nu }_{{\hat{a}}}]\approx \cos (\hat{\psi })^{SS} + \eta _{\mu \nu } \delta \ell ^{\mu }E^{\nu }_{{\hat{a}}} + h^{GW}_{\mu \nu } \ell ^{\mu } E^{\nu }_{{\hat{a}}}. \end{aligned}$$

However, this form of the observation equation explicitly depends on the observer orientation, i.e., on satellite attitude, with the drawbacks in relation to attitude errors heavily affecting the error budget of the measurements.

### The astrometric gravitational wave shift

The four tangent vector to a null geodesic satisfies the well known conditions:

24 $$\begin{aligned} k^\alpha \nabla _\alpha k^\beta =0, \qquad k^\alpha k_\alpha =0, \end{aligned}$$

$\nabla _\alpha$ being the covariant derivative associated with the spacetime metric. The decomposition of the photon 4-momentum with respect to an observer $u^{\alpha }$ implies that the trajectory is parametrized by $\sigma$ such that

25 $$\begin{aligned} {\bar{k}}^\alpha = -\frac{k^\alpha }{(u|k)}=\frac{{\textrm{d}} x^\alpha }{{\textrm{d}} \sigma }, \end{aligned}$$

and Eq. (24) becomes

26 $$\begin{aligned} {\bar{k}}^\alpha \nabla _\alpha {\bar{k}}^\beta =-\frac{{\textrm{d}}\ln {[-(u|k)]}}{{\textrm{d}}\sigma }{\bar{k}}^\beta \,, \end{aligned}$$

which is related to the affine parameter $\lambda$ by $\mathrm d \sigma =-(u|k) \mathrm d \lambda$.

It is easy to check that in the case of a static observer

27 $$\begin{aligned} \frac{{\textrm{d}} \ln {[-(u|k)] }}{{\textrm{d}}\sigma }=\ell ^\alpha \ell ^\beta k_{\alpha \beta }-\ell ^\alpha a_\alpha =-\ell ^\alpha \ell ^\beta \theta _{\alpha \beta }- \ell ^\alpha a_\alpha , \end{aligned}$$

where the two spatial fields coming from the splitting of the covariant derivative of *u*, i.e., $\nabla _{\beta }u^\alpha =-a^\alpha u_\beta -k^{\alpha }{}_{\beta }$, the acceleration vector $a^\alpha$ and the kinematical tensor $k^{\alpha }{}_{\beta }=\omega ^{\alpha }{}_{\beta } -\theta ^{\alpha }{}_{\beta }$ are expressed as a combination of the vorticity and expansions of the congruence of curves related to fiducial observers $u^{\alpha }$. Thus, the geodesic equation transforms into

28 $$\begin{aligned} \frac{{\textrm{d}} {\bar{k}}^\alpha }{{\textrm{d}} \sigma }+ \Gamma ^\alpha {}_{\mu \nu }{\bar{k}}^\mu {\bar{k}}^\nu -\left[ \ell ^\mu \ell ^\nu \theta _{\mu \nu }+ \ell ^\mu a_\mu \right] {\bar{k}}^\alpha =0, \end{aligned}$$

or

29 $$\begin{aligned} \frac{{\textrm{d}} \ell ^\alpha }{{\textrm{d}} \sigma }+ & {} \Gamma ^\alpha {}_{\mu \nu } \ell ^\mu ( \ell ^\nu +u^\nu )+a^\alpha -k^\alpha {}_\sigma \ell ^\sigma -\left[ \ell ^\mu \ell ^\nu \theta _{\mu \nu }+ \ell ^\mu a_\mu \right] (\ell ^\alpha +u^\alpha )=0\,, \end{aligned}$$

for the unknown local line-of-sight $\ell ^\alpha$. In case of static observers, the kinematical fields reduce to

30 $$\begin{aligned} a^i=\partial _0 h_{0i}-\frac{1}{2}\partial _i h_{00} =h_{0i,0}-\frac{1}{2} h_{00,i},\qquad \theta _{ij}=\frac{1}{2} h_{ij,0},\qquad \omega _{ij} =-h_{0[i,j]}. \end{aligned}$$

Note that considering the metric as an approximate solution of the Einstein field equation composed of a background part plus a GW perturbation, also the affine coefficients can be split respectively into two parts

31 $$\begin{aligned} \Gamma ^\alpha {}_{\mu \nu } = \frac{1}{2} g^{\alpha \rho }\left( g_{\rho \beta ,\gamma }+g_{\rho \gamma ,\beta }-g_{\beta \gamma ,\rho }\right) \approx \Gamma ^{\alpha (SS)}_{\beta \gamma } + \Gamma ^{\alpha (GW)}_{\beta \gamma }, \end{aligned}$$

at the first order of the perturbation. The same for the parameter sigma

32 $$\begin{aligned} d\sigma = - (u|k) d\lambda = - [ (g_{\alpha \beta }^{(SS)} + h_{\alpha \beta }^{(GW)}) \, u^{\alpha } k^{\beta } ]d\lambda . \end{aligned}$$

The TT gauge choice implies that the second term of equation (32) does not contribute.

All of the above implies the possibility again to split Eq. (29) into the Solar System part plus the GW one, thus it allows to integrate separately each term. As a matter of fact, at the order of $\epsilon ^4$, it is possible to isolate from Eq. (29) the contribution of the GW part and obtain

33 $$\begin{aligned} \frac{d \delta \ell ^i}{d \sigma } \approx - \frac{1}{2} \ell ^j_{_0} \ell ^k_{_0} (2 h^{GW}_{ij,k} - h^{GW}_{jk,i}) - \ell ^j_{_0} h^{GW}_{ij,0} + \frac{\ell ^j_{_0} \ell ^k_{_0} \ell ^i_{_0}}{2} h^{GW}_{jk,0}. \end{aligned}$$

Since $d\sigma = d\lambda + O(\epsilon ^2)$ and assuming for the photon trajectory $x^0(\sigma ) = x^0_{obs} + \sigma + O(\epsilon ^2)$ and $x^i(\sigma ) = x^i_{obs}+ \ell ^i_{ _0} \sigma + O(\epsilon ^2)$, the argument of $h^{GW}$ becomes:

34 $$\begin{aligned} {\tilde{k}}_{\alpha } x^{\alpha } = {\tilde{k}}_0 \sigma (1 + p \cdot \ell _{_0}) + \tilde{\psi }+ O(\epsilon ^2) \end{aligned}$$

where $\tilde{\psi }= {\tilde{k}}_{\alpha } x_{obs}^{\alpha }$ can be considered a phase term. Then,

35 $$\begin{aligned} h_{ij,0}^{GW} = \frac{1}{(1 + p \cdot \ell _{ _0} )} \frac{dh^{GW}}{d \sigma }, \,\,\,\, h_{ij,k}^{GW} = \frac{ p^k}{ (1 + p \cdot \ell _{ _0}) } \frac{dh^{GW}}{d \sigma }. \end{aligned}$$

Via a direct integration of Eq. (33) we easily obtain the gravitational shift of the local direction:

36 $$\begin{aligned} \int ^{\sigma _*}_{\sigma _{obs}} d \delta \ell ^i = \frac{ \ell ^i_{_0} + p^i}{2 (1 + p \cdot \ell _{_0})} \ell ^j_{_0} \ell ^k_{_0} \int ^{\sigma _*}_{\sigma _{obs}} d h_{jk}^{(GW)} (\sigma ) - \frac{ \ell ^j_{_0}}{2} \int ^{\sigma _*}_{\sigma _{obs}} d h_{ij}^{(GW)} (\sigma ) + O(\epsilon ^5), \end{aligned}$$

which coincides with the result in Ref.^14^ when the distance to the stellar source is many gravitational waves away, namely the detection occurs in the far-away wave zone.

Any variation induced by the GW on the orthonormal basis is in principle absorbed as second order effects, or in the calculations if proportional to the GW strain. In conclusion, the derivation of Eqs. (9) and (12) were obtained by solving the geodesic for the null four vector with respect to its affine parameter and taking into account the kinematical geometrical proprieties of the congruence of the fiducial observers and their variations, as expressions presented here show, consistently with the GR theory of measurement and the results of the cited Authors.

## Data Availability

All data generated or analysed during this study are included in this published article.
